# Eye Disorders in Old People

**DOI:** 10.5539/gjhs.v5n1p79

**Published:** 2012-11-06

**Authors:** Khalaj M., Barikani A., Ghasemi H.

**Affiliations:** 1 Department of Ophthalmology, Qazvin University of Medical Sciences, Qazvin, Iran; 2 Department of Community Medicine, Qazvin University of Medical Sciences, Qazvin, Iran; 3 General Physician, Qazvin University of Medical Sciences, Qazvin, Iran

**Keywords:** refractory error, myopia, hyperopia, astigmatism, amblyopia, diabetic retinopathy, cataract, glaucoma

## Abstract

**Background:**

Visual impairment is one of a major public health problem among elderly people.

**Object:**

Aim of this study was determining the prevalence of visual impairment in median and old peoples in Qazvin (Iran).

**Method:**

In this cross sectional study, with a simple random sampling, 446 patients older than 5o years who were referred to outpatient ophthalmology clinics at Avecina hospital of Qazvin (a province of Iran) in 2010 were enrolled. Participants first complete a questionnaire with 25 questions toward demographic and past medical history and then were examined by ophthalmologist. These examinations includes direct and indirect ophthalmoscopy, slit lamp examination, measurement of uncorrected visual acuity and visual acuity with current glasses, lensometery of the previous glasses, refraction with and without the use of cycloplegic and determining the best corrected visual acuity. All slit lamp examinations were performed by the same ophthalmologist. Data were analyzed with SPSS16 with use of Chi – Square test with Pvalue <0.05.

**Results:**

In this study 446 patients were examined that 54.7% were male. Mean age of study population was 62+-9.3 years old. 96.4% of participants had refractory disorder. Prevalence of myopia, hyperopia and astigmatism were 33.6%, 45.9% and 16.8% respectively. Of patients 17.4% had diabetes. Of participants 28.9% had temporal headache, 37% red eye, 41.2% flashing, 27.3% and 28% had dryness and discharge of eye respectively. 31.1% of participants had eyelide problem, 4.7% Color Vision Deficiency (CVD) and 3.8% had family history of CVD. Of total 4.5% had glaucoma, 3.3% macular degeneration and 21.7% had hypertension. 0.6% of population had macular degeneration, 0.4% of population had glaucoma Of 892 eyes (446 individuals), 36.2% had visual acuity less than 7/10, 1.7% light perception (LP) and 0.22% no light perception (NLP) and 2.7% finger count.

**Conclusion:**

Refractory errors, cataract and amblyopia were most important eye disorders in older people in Qazvin.

## 1. Introduction

Visual impairment is a major public health problem among elderly. Over 285 million people in the world are visually impaired, of whom 39 million are blind and 246 million have moderate to severe visual impairment (WHO, 2010). It is predicted that without extra interventions, these numbers will rise to 75 million blind and 200 million visually impaired by the year 2020 (WHO, 2011). WHO (World Health Organization) also estimated that about 80% of blindness around the world is avoidable and 90% of the world’s blind live in a developing country (WHO, 2010). Among persons who are blind worldwide 58% are age 60+ and 32% are ages 45-59. The main causes of blindness are cataract (47.8%), glaucoma (12.3%) and age related macular degeneration (8.7%). Other causes include corneal opacity (5.1%), diabetic retinopathy (4.8%), childhood blindness (3.9%), trachoma (3.6%), and onchocerciasis (0.8%) (WHO, 2010). The prevalence of visual impairment increased rapidly with age among all racial and ethnic groups, particularly among people older than 75 years (National Institutes of Health, 2008). Cases of macular degeneration are expected to double by 2050, from 9.1 million to 17.8 million for aged 50 years or older (Rein et al., 2009). Cases of diabetic retinopathy among people older than 65 or older are expected to quadruple by 2050 from 2.5 million to 9.9 million (Saaddine et al., 2008). With increasing of chronic disease in developed and developing country, prevalence of visual loss is increased. In all developing countries the death and disability from chronic disease now exceeds that from communicable disease comprising 49% compaired with about 40% for communicable disease and 11% for injuries (Lopez et al., 2006). Many studies showed that visual loss is associated with higher prevalence of chronic health conditions (Crews et al., 2006), death (Lee et al., 2002), falls and injuries (Ivers et al., 2000), depression and social isolation (Jones et al., 2009; Horowitz et al., 2003; Rein et al., 2006). The trend of increasing mean age of people in Iran has been started. The mean age of population in Iran would be increased 10 years between 2006-2026 (Mirzaie et al., 2007). Iranian old peoples such as other developing countries have fronted with many of chronic disease and injuries that could be affected on visual power and finally quality of life. A few studies are conducted on visual impairment on old peoples in Iran. Therefore we designed this study with the aim of determining the prevalence of visual impairment in median and old peoples for early detection, prevention, treatment and rehabilitation.

## 2. Method

In this cross sectional study, with a simple random sampling, 446 patients older than 5o years old who were referred to outpatient ophthalmology clinics at Avecina hospital of Qazvin (a province of Iran) in 2010 were enrolled. This clinic is one of the main academic medical institutions in Qazvin that provided comprehensive health care service for residents of Qazvin city and suburb and therefore appropriate for population based studies. Approval for this study was obtained from Qazvin University of Medical Sciences. Participants first complete a questionnaire with 25 questions toward demographic and past medical history and then were examined by ophthalmologist. These examinations includes direct and indirect ophthalmoscopy, slit lamp examination, measurement of uncorrected visual acuity and visual acuity with current glasses, lensometery of the previous glasses, refraction with and without the use of cycloplegic and determining the best corrected visual acuity. Visual acuity was determined by using Snellen chart. To evaluate visual acuity, the person was asked to read or guess the letters in the upper most line, if a person was not to able to see the first line, finger count, hand motion and light perception were used. Visual acuity of participants was measured separately for each eye according to modified from the international classification of disease, 9^th^ rev, clinical modification. According to this, visual acuity in range of 20/10- 20/25 and 20/28- 20/60 is normal and near normal respectively, visual acuity between 20/70- 20/160 and 20/200- 20/400 is moderate and severe low vision respectively and visual acuity less than 20/400 is blindness (American Optometric Association, 2007).

Myopia was defined as an SE (spherical equivaleat) of -0.5D or worse. An SE of +0.5D or worse for non cycloplegic refraction and SE of +2D or worse for cycloplegic refraction was used to define hyperopia, and astigmatism was defined as cylinder power of at least 0.75D (Attebo et al., 1999; Wong et al., 2000; Wu et al., 1999; Gwiazda et al., 1984; Saw et al., 2003). Amblyopia was defined as best corrected visual acuity less than 10/10 or a difference between the two eyes of at least 2/10. Glaucoma was diagnosed on the basis of statistical abnormality of the vertical CDR (cup disc ratio) combined with an abnormal visual field test or in subjects with advanced glaucoma who could not complete field testing a grossly abnormal CDR. If it was not possible to examine the optic disc and the subject was blind, glaucoma was diagnosed on the basis of intraocular pressure. All patients Know to have diabetes (FBS ≥ 126mg/dl) underwent an eye examination by indirect ophthalmoscope to check for any signs of diabetic retinopathy through dilated pupils by +78 lens. All slit lamp examinations were performed by the same ophthalmologist. Data were analyzed with SPSS16 with use of Chi – Square test with P Value <0.05.

## 3. Results

In this study 446 patients were examined that 54.7% were male. Mean age of study population was 62+-9.3 years old. 96.4% of participants had refractory disorder, that of them 33.6% had myopia (Table 1).

**Table 1:** Frequency and percent of age, sex, family history of participants (Qazvin, 2010) (N=446)

	N	%
***Age***		
50-60	238	53.4
60-70	124	27
70-80	77	17.3
80-90	5	1.1
>90	2	0.4
***Sex***		
Female	202	45.3
Male	244	54.7
***Refractory errors***		
Myopia	150	33.6
Hyperopia	205	45.9
Astigmatism	75	16.8

26.1% of them had low vision in range of moderate to severe (Table 2).

**Table 2:** Frequency and percent of visual impairment, low vision, and blindness by sex and age based on presenting visual acuity (Qazvin, 2010) (N=446)

	normal vision		low vision(moderate and severe)		P
Female	148	33.9%	50	11.5%	0.6
Male	175	40.0%	64	14.7%	
Total	323	73.9%	114	26.1%	

37% of patients had family history of vision loss, 0.6% cataract and 17.9% diabetes. Of patients 17.4% had diabetes. Of participants 28.9% had temporal headache, 37% red eye, 41.2% flashing, 27.3% and 28% had dryness and discharge of eye respectively. 31.1% of participants had eyelide problem, 4.7% Color Vision Deficiency (CVD) and 3.8% had family history of CVD. Of total 0.4 % had glaucoma, 0.6% macular degeneration table 3) and 21.7% had hypertension.

**Table 3:** Frequency percent of eye disorders in old man and woman in Qazvin (2010) (N=446)

Disorders	Female	Male	P
Cataract	4.3	7.6	0.09
Retinopathy	2.5	1.3	0.08
Aphakia	0.9	1.8	0.2
Ptosis	0.4	0.7	0.5
Anisometria	0.4	0.7	0.5
Retinal Detachment	0.9	0.7	0.3
Glaucoma	0.2	0.2	0.7
Aniso cornea	0.0	0.4	0.2
Hemorraghy	0.0	0.4	0.2
Macular degeneration	0.4	0.2	0.4
Ectropion	0.7	0.0	0.09
Amblyopia	18.4	20.2	0.2

Of 11.9% participant that had cataract 4.9% were in 50-59 years age group (Table 4).

**Table 4:** Frequency percent of eye disorders with age in elderly people in Qazvin (2010) (N=446)

	50-59 (N=220)	60-69 (N=132)	70-79 (N=76)	≥80 (N=15)	Total (N=446)	P
Cataract	4.9	2.9	3.4	0.7	11.9	0.1
Retinopathy	2	1.3	0.4	0	3.8	0.8
Aphakia	1.3	1.1	0.2	0	2.7	0.7
Ptosis	0	0.7	0.2	0.2	1.1	0.001
Anisometria	0.7	0.2	0.2	0	1.1	0.9
Shalazion	0	0	0	0.4	0.4	0.001
Retinal Detachment	0.7	0.7	0.2	0	1.6	0.9
Glaucoma	0	0.2	0.2	0	0.4	0.6
Aniso cornea	0	0.4	0	0	0.4	0.3
Hemorraghy	0	0.2	0	0	0.4	0.6
Macular degeneration	0.2	0.2	0.2	0	0.6	0.9
Ectropion	0.2	0.4	0	0	0.7	0.7
Amblyopia	17.3	12.6	6.7	2	38.6	0.1

Of 892 eyes (446 individuals), 36.2% had visual acuity less than 7/10, 1.7% light perception (LP) and 0.22% no light perception (NLP) and 2.7% finger count.

## 4. Discussion

Visual problem is the most common eye disorders especially in old people. One of the most important eye problems in our study was dryness of eye, similar to other studies (Steven et al., 2009; Kaercher et al., 2008).

Dry eye is a very common condition that is characterized by a disturbance of the tear film. This abnormality may result in disruption of the ocular surface and causing a variety of symptoms and signs. This disorder affects especially population older than 40 years of age. With increasing of age, tear production by the tear glands has been decreased. Other causes of poor production of tears are hormonal changes and autoimmune disease. Patients with dry eye may experience scratchy, burning or itching, redness, blurred vision, foreign body sensation and light sensitivity (Steven et al., 2009; King-Smith et al., 2009; Foulks et al., 2008). Another eye problems in our study were refractory errors; hyperopia (45.9%), myopia (33.6%) and astigmatism (16.8%). The significant proportion of visual impairment and blindness is because of refractory errors (Munoz et al., 2002; Dandona et al., 2001; VanNewkirk et al., 2001). The prevalence of refractory errors varies according to age and ethnic groups. Some population based studies were conducted in developing and developed countries in Asia (Dandona et al., 2002; Dandona et al., 1999; Murthy et al., 2002; Lin et al., 2001; Wong et al., 2000), but a few studies were conducted in elderly population. In a study in Tehran (capital of Iran) 58.9% of visual impairment is due to cataract and refractory errors that are easily curable (Fotouhi et al., 2004).

The population of old people is rapidly growing in Iran. With increasing of this population, age related eye problems have been increased and health care system must notice this condition for prevention planning. Retinal disorder such as retinal detachment (1.6%), retinopathy (3.8%), macular degeneration (0.6%) was another eye problem in population study. Diabethic retinopathy is one of old aged people disease that started with decrease of vision and follow them cause retinal hemorrhagy (Khalaj et al., 2008).

About 10.2 million US adults 40 years and older known to have DM (diabetes mellitus) and crude prevalence rates for retionopathy was 40.3% and in general population this rate was 3.4% that similar to our study (American Medical Association, 2004). In other study in Tehran (Iran) diabetic retionopathy in diabetic patients was 37% (Javadi et al., 2009). Cataract was one of the most eye disease among people older than 50 years old in our study. In a epidemiologic study in America the prevalence of cataract in population older than 40 years was 17.2% and women significantly had higher prevalence than men but in a study in Tehran 22.7% of population older than 40 years had cataract and men had lower prevalence than women (Hashemi et al., 2009). In this study we showed that eye disorders were not different in age groups, only ptosis was seen more in higher age groups. In our study 0.6% of population had macular degeneration, compare to 1.47% in prevalence study in America. The prevalence of macular degeneration increased with age and is a leading cause of blindness in Australia (Mitchell et al., 2005; VanNewkirk et al., 2000).

In our study 0.4% of population had glaucoma. The overall prevalence of open angle glaucoma in the US population 40 years and older was 1.86%. In other study prevalence was 2.1% (Klein, 2004).

Other prevalent problem in study population was amblyopia (18.2% and 20.2% in female and male). Amblyopia is a frequent cause of unilateral or bilateral blindness. The prevalence of amblyopia ranged from 2% to 4% of population (Chang, Tsai, & Sheu, 2007).

In a study in Iran prevalence of amblyopia in schoolchildren was 1.9% in girls and 1.7% in boys (Faghihi, Ostadimoghaddam, & Yekta, 2011). If amblyopia don’t treated in children it could lead to visual impairment or blindness. Amblyopia was not found to be statistically different by gender, similar to Australians study. In our study prevalence of myopia, hyperopia and astigmatism were 33.6%, 45.9% and 16.8% respectively, that similar to other studies (Wang et al., 1994; Vitale et al., 2008; Wu et al., 1999; Attebo et al., 1999; Shufelt et al., 2005).

Refractive errors are prevalent problem that affect all aged groups. In many studies prevalence of myopia was reported to be 15% (Attebo et al., 1999) to 51% (Gupta, 2008) and hyperopia 3.6% to 63.8% (Hashemi et al., 2009; Vitale, 2008).

In a study in Mashhad (Iran) this prevalence were 17%, 41.3% and 5.6% respectively (Ostadimoghaddam, 2011).

The results of present study showed that, refractory errors, amblyopia, cataract and diabethic retinopathy was prevalent eye problems in old people in Qazvin. Population of elderly persons in Iran presents a growing challenge to primary health care systems.

Many studies about impact of eye disorders on quality of life (QOL) showed that elderly persons with low vision had low QOL and high mortality (Nishinaga et al., 2007; Jacobs et al., 2005; Nirmalan et al., 2005).

Center for disease control and prevention (CDC) reported that vision disability is one of the top 10 disabilities among adults aged 18 years and older. Although aging is unavoidable but we can modify many of risk factors such as smoking, ultra violate light exposure, wrong diet and life style to prevent or slowing the trend of eye disorders in elderly people to increasing QOL (CDC, 2009). Although health care program for elderly people integrated in Iranian primary health care but attention to eye disorders and vision loss is not enough. This problem may be because of wrong understand of decision makers from eye disorders prevalence. In this study we showed that eye disorders in elderly people were considerable, and if neglected can lead to health problems and decrease of QOL in growing elderly people. Therefore decision maker in health system must notice this fact for planning preventive programs in order to health promotion.
